# Associations between insulin and glucose concentrations and anthropometric measures of fat mass in Australian adolescents

**DOI:** 10.1186/1471-2431-10-58

**Published:** 2010-08-11

**Authors:** Elizabeth Denney-Wilson, Christopher T Cowell, Anthony D Okely, Louise L Hardy, Robert Aitken, Timothy Dobbins

**Affiliations:** 1 University of New South Wales Research Centre for Primary Health Care and Equity, Sydney, 2052, Australia; 2 Institute of Endocrinology and Diabetes, Children's Hospital at Westmead, Westmead, 2145, Australia; 3 Child Obesity Research Centre and Faculty of Education, University of Wollongong, Wollongong, 2522, Australia; 4 Physical Activity, Nutrition and Obesity Research Group, University of Sydney, Sydney, 2006, Australia; 5 Lowy Cancer Research Centre, University of New South Wales, Sydney, 2052, Australia

## Abstract

**Background:**

One of the most serious, yet common co-morbidities of obesity is insulin resistance, which if untreated may progress to type 2 diabetes. This paper describes the insulin and glucose concentration distributions, the prevalence of elevated insulin, the associations between insulin and body mass index (BMI), waist circumference, waist-to-height ratio (WHtR) and fat mass index in a representative sample of Australian adolescents.

**Methods:**

Cross-sectional population-based study of adolescent boys and girls (N = 496, mean age 15.3 years) attending schools in metropolitan Sydney, Australia. Fasting venous blood collected and analysed for insulin and glucose concentrations. Height, weight, waist circumference measured, BMI and waist-to-height ratio calculated. Pubertal status self-reported.

**Results:**

Glucose concentrations were normally distributed and were not associated with adiposity. Insulin concentrations were distributed logarithmically, were higher among girls than boys overall and within the same ranges of BMI and waist circumference, but were lower among girls than boys within the same ranges of fat mass adjusted for height. The prevalence of elevated insulin concentration (defined as > 100 pmol/L) was 15.9% and 17.1% among boys and girls, respectively. Correlations between insulin concentration and BMI, waist circumference, WHtR and fat mass adjusted for height were 0.53, 0.49, 0.51 and 0.55, among boys, respectively, and 0.35, 0.40, 0.42 and 0.34, among girls, respectively.

**Conclusions:**

Elevated insulin is highly correlated with adiposity in adolescents. BMI and WHtR are simple measures that can be used to identify young people who should be screened for insulin resistance and other co-morbidities.

## Background

Overweight and obese children and adolescents are at increased risk for the development of a range of chronic, debilitating health problems [1]. Just as the prevalence of obesity among children and adolescents is increasing, so is the prevalence of type 2 diabetes, the metabolic syndrome, fatty liver disease and cardiovascular disease [2]. However, it is not clear which young people should be screened for co-morbidities, or which anthropometric measure provides the best guidance to clinicians as to the level of risk and the need for further investigation.

Very little is known about the population prevalence and distribution of adverse concentrations of insulin, the nature of the distributions of glucose and insulin concentrations and the nature of the associations between glucose and insulin concentrations and adiposity among young people. There have been three reports on glucose and insulin concentrations based on population samples. Allard et al. [3] described insulin and glucose concentration distributions among 9, 13 and 16 year old Quebec students (n = 2,244), Wennlöf et al. [4] did the same for 9 and 15 year old Swedish students (n = 1137) and Thorsdottir et al. reported insulin concentration among 9 and 15 year old Icelandic students [5].

Several studies have assessed the associations between adiposity and glucose and insulin concentration. Steinberger et al. found that fasting insulin values were significantly higher among obese compared with non-obese adolescents [6]. Arslanian and Suprasongsin found that fasting insulin concentration was significantly correlated with percentage body fat (%BF) among 20 peri-pubertal young people [7]. Misra et al., found that, among Asian-Indian 14-18 year olds fasting insulin correlated significantly with BMI, %BF, waist circumference and with skinfold thicknesses [8]. Wennlöf et al. reported that insulin concentration was only elevated in the highest decile of BMI among 15 year old Swedish boys and girls [4]. Garnett et al found that adolescents with a WHtR of greater than 0.5 were significantly more likely to have cardiovascular risk factors than those with a lower WHtR [9] while data from the Bogalusa Heart Study suggests that BMI and WHtR are both suitable anthropometric measures to identify children at risk of cardiovascular disease [10]. Finally, Thorsdottir et al. reported that the values of different measures of adiposity increased significantly across quartiles of insulin concentration [5]. These studies have various limitations, including: combining data for boys and girls; small sample sizes; combining young people in various stages of pubertal development; or using only a single measure of adiposity.

The purposes of this paper were to describe the population distributions of insulin and glucose concentration, the prevalence of elevated insulin concentrations and the associations between insulin concentration and BMI, waist circumference, waist-to- height ratio (WHtR) and fat mass index for boys and girls separately. The data were drawn from a representative sample of Grade 10 students (age range 14.3 - 17.1 years) living in the Sydney (Australia) metropolitan area (population = 4.2 Million).

## Methods

The methods employed in the NSW Schools Physical Activity and Nutrition Survey, 2004 (SPANS 2004) have been described in detail elsewhere [11]. The study was approved by the University of Sydney Human Research Ethics Committee and the Government, Catholic and Independent school sectors and informed consent by the students and their carers was required for participation.

SPANS 2004 was a representative population survey of students attending kindergarten (Grade K) and Grades 2, 4, 6, 8 and 10 in primary and secondary schools in NSW, Australia. The data were collected from February to May 2004. Blood samples for the biomarker study reported here were only collected from Grade 10 students attending schools in the Sydney metropolitan area.

Standard methods were used to measure height, weight and waist circumference [11]. BMI was calculated (kg/m^2^) and categorised as not overweight/obese, overweight or obese using IOTF definitions [12]. Waist-to-height ratio was calculated by dividing waist circumference (in cm) by height (in cm). Overnight fasting blood samples were analysed by an accredited laboratory. There is currently no consensus on a definition of "elevated" fasting insulin concentration among adolescents, however one of the authors (CTC), a pediatric endocrinologist, identified the value of fasting insulin at which clinicians would pursue further investigations and consider treatment as > 100 pmol/L. Glucose concentrations greater than 110 mg/dL were defined as high based on the American Academy of Pediatrics' National Cholesterol Education Program definition [13].

Fat mass was estimated by calculating percentage body fat (%BF) using the regression equations of Taylor et al. [14] then calculating fat mass (kg) as %BF * body mass (kg). Height correlated significantly with fat mass among boys (r = 0.211, P < 0.001) and girls (r = 0.215, P = 0.002). The fat mass index (FMI) was therefore calculated as fat mass (kg)/height (m)^2^. The FMI did not correlate significantly with height among boys (r = 0.086, P = 0.142) or girls (r = 0.070, P = 0.317).

Based on the pubertal ratings as determined by Tanner [15], boys were asked to self-report their stage of pubic hair development, while girls were asked to report their stage of breast development and age of menarche.

### Data analysis

Data were analysed using SAS Version 9.1. Glucose and insulin concentration distributions were characterised and compared by sex and BMI category. Where data were log-normally distributed, values were log-transformed prior to analyses and reported as geometric rather than arithmetic means. Overall BMI, waist circumference, waist-to-height ratio and FMI quintiles were estimated and mean insulin concentrations calculated by quintile for boys and girls separately. Linear and logistic regression models adjusted for the survey design were used to test for differences in concentrations across BMI categories and between sexes.

## Results

### Characteristics of the sample

The characteristics of the sample are shown in Table 1. There was no significant difference in the prevalence of overweight and obesity combined among all Grade 10 students and those students who provided blood; 26.6% and 27.6%, respectively, among boys and 18.9% and 19.4%, among girls, respectively. Furthermore, the proportions in each BMI category were not significantly different between those who were invited to give blood and declined and those who agreed to give blood for boys (Χ_2_^2 ^= 0.51, P = 0.8) or girls (Χ_2_^2 ^= 0.73, P = 0.7). More than 90% of boys and girls in this study were in the latter stages of puberty, and mean insulin values did not vary consistently across Tanner stages within BMI categories.

**Table 1:** Participation rates and characteristics of the sample

	Boys	Girls
**n (%)**	290 (58.6)	205 (41.4)
**Participation rate (%)**	40.7	39.4
**Age (years)**		
mean ± SE	15.4 ± 0.03	15.4 ± 0.03
range	14.3 - 17.1	14.6 - 16.9
**Height (cm)**		163.9 ± 0.55
mean ± SE	173.5 ± 0.32	163.7
median	173.7	148.0 - 178.9
range	153.0 - 192.0	
**Weight (kg)**		
mean ± SE	66.3 ± 1.14	57.9 ± 1.03
median	64.0	56.8
range	38.7 - 139.4	32.1 - 105.8
**BMI (kg/m^2^)**		
mean ± SE	21.9 ± 0.34	21.5 ± 0.32
median	21.1	20.8
range	15.3 - 41.4	14.0 - 37.5
**BMI categories (%)**		
not overweight/obese	72.4	80.5
overweight	21.0	15.1
obese	6.6	4.4
**Waist circumference (cm)**		
mean ± SE	71.9 ± 0.92	65.8 ± 0.91
median	69.8	64.7
range	54.1 - 109.1	48.9 - 101.8
**Waist to Height Ratio (cm/cm)**		
mean ± SE	0.41 ± 0.005	0.40 ± 0.005
median	0.40	0.39
range	0.33 - 0.64	0.32 - 0.63
**Fat mass (kg)**		
mean ± SE	14.3 ± 0.78	17.8 ± 0.80
median	11.6	15.9
range	4.0 - 88.4	4.8 - 66.8
**FMI (kg/m^2^)**		
mean ± SE	4.7 ± 0.25	6.6 ± 0.29
median	3.8	5.7
range	1.5 - 26.2	2.1 - 25.9

### Population distributions of glucose concentration

Glucose concentration was normally distributed among boys and girls and within each BMI category. Table 2 shows the mean (± SE), the percentile values for the whole sample and for each BMI category, for boys and girls. For the whole sample, the values of glucose concentration ranged from 2.5-6.4 mmol/L and 3.2-5.5 mmol/L among boys and girls, respectively. A value of 6.4 mmol/L in the boys' overweight category appeared to be atypically high. The distributions of glucose concentration were similar for boys and girls. Among boys, glucose concentration increased only slightly across BMI categories and among girls the distributions were similar across BMI categories. Among girls, atypically high values of 5.5 mmol/L in the healthy BMI category and 5.4 mmol/L in the overweight BMI category were noted.

**Table 2:** Sample distributions of glucose (mmol/L) for boys and girls, combined and in not overweight/obese (NOO), overweight (Ow) and obese (Ob) BMI categories

Boys					Girls				
	All	NOO	Ow	Ob	All	NOO	Ow	Ob	
**n**	290	210	61	19	206	165	31	9	**n**
centile									centile
0	2.5	2.5	3.2	3.4	3.2	3.2	3.7	4.0	0
5	3.9	4.0	3.7	3.4	4.0	4.0	3.9	4.0	5
10	4.1	4.2	4.0	4.0	4.1	4.1	4.2	4.0	10
15	4.2	4.2	4.3	4.2	4.2	4.2	4.2	4.1	15
25	4.4	4.4	4.5	4.7	4.3	4.4	4.3	4.4	25
30	4.5	4.4	4.6	4.7	4.4	4.4	4.3	4.4	30
40	4.6	4.5	4.7	4.8	4.4	4.5	4.4	4.4	40
median	4.7	4.6	4.7	4.8	4.5	4.5	4.6	4.4	median
60	4.8	4.7	4.8	4.9	4.6	4.6	4.6	4.6	60
70	4.9	4.8	4.9	5.0	4.7	4.7	4.8	4.9	70
75	4.9	4.9	4.9	5.0	4.7	4.7	4.8	4.9	75
85	5.1	5.1	5.1	5.2	4.8	4.8	4.8	4.9	85
90	5.2	5.2	5.1	5.3	4.9	4.9	4.9	4.9	90
95	5.3	5.3	5.2	5.7	5.1	5.1	4.9	4.9	95
100	6.4	6.1	6.4	5.7	5.5	5.5	5.4	4.9	100
mean	4.64	4.62	4.67	4.78	4.52	4.52	4.52	4.51	mean
SE	0.03	0.03	0.08	0.09	0.03	0.03	0.07	0.11	SE

### Population distributions of insulin concentration

Table 3 shows the percentile values of insulin concentration for the whole sample and all BMI categories, for boys and girls. The values of insulin concentration ranged from 13.9-243.1 pmol/L and 13.9-284.7 pmol/L for boys and girls, respectively. The distributions were similar for boys and girls, although the values were slightly higher among girls across the distribution, up to approximately the 85^th ^percentile. Among boys, atypically high values of 222 pmol/L in the non-overweight/obese BMI category and 215 pmol/L in the overweight BMI category were noted. Among girls, one atypically high value of 284 pmol/L in the non-overweight/obese BMI category were also noted. There was a statistically significant difference for males (F = 50.84, p < 0.0001) and females (F = 12.31, p = 0.005). Geometric mean insulin concentrations were statistically significantly higher in overweight (t = 6.59, p < 0.0001) and obese males (t = 9.21, p < 0.0001) than in healthy males. A similar pattern was seen in overweight (t = 2.44, p = 0.0258) and obese (t = 3.22, p = 0.0051) females.

**Table 3:** Sample distributions of insulin (pmol/L) for boys and girls, combined and in not overweight/obese (NOO), overweight (Ow) and obese (Ob) BMI categories

Boys					Girls				
	All	NOO	Ow	Ob	All	NOO	Ow	Ob*	
**n**	290	210	61	19	206	165	31	9	**n**
**centile**									**centile**
0	13.9	13.9	34.7	48.6	13.9	13.9	34.7	41.7	0
5	27.8	27.8	41.7	48.6	34.7	27.8	41.7	41.7	5
10	27.8	27.8	48.6	48.6	34.7	34.7	48.6	41.7	10
15	34.7	27.8	48.6	62.5	41.7	41.7	48.6	69.5	15
25	41.7	34.7	55.6	104.2	48.6	48.6	55.6	69.5	25
30	41.7	41.7	62.5	104.2	48.6	48.6	55.6	69.5	30
40	48.6	41.7	69.5	132	55.6	55.6	62.5	97.2	40
median	62.5	48.6	76.4	159.7	62.5	62.5	90.3	111.1	median
60	69.5	62.5	90.3	180.6	76.4	69.5	118.1	159.7	60
70	79.9	69.5	111.1	187.5	90.3	83.3	125	159.7	70
75	90.3	76.4	118.1	187.5	97.2	90.3	132	159.7	75
85	118.1	90.3	138.9	229.2	118.1	104.2	166.7	166.7	85
90	138.9	104.2	152.8	229.2	132	118.1	173.6	166.7	90
95	159.7	125	159.7	243.1	159.7	132	187.5	166.7	95
100	243.1	222.2	215.3	243.1	284.7	284.7	194.5	166.7	100
Mean^1^	61.2	52.47	81.52	133.51	67.78	62.97	88.35	104.91	Mean^1^
SE^2^	0.05	0.05	0.05	0.09	0.05	0.05	0.12	0.2	SE^2^

1 Geometric mean

2 Standard error of the natural log of the geometric mean

### Prevalence of elevated insulin concentrations

Overall, the prevalences of elevated insulin concentrations > 100 pmol/L were 19.3% and 22.4% among boys and girls, respectively. Among boys the prevalences of elevated insulin concentrations in the non-overweight, overweight and obese BMI categories were 7.1%, 29.5% and 68.4%, respectively, and among girls, were 10.9%, 41.9% and 44.4%, respectively. Statistically significant overall differences were found for males (Χ^2 ^= 59.4, p < 0.0001) and females (Χ^2 ^= 21.9, p < 0.0001). Specifically, overweight (Χ^2 ^= 12.7, p = 0.0004) and obese (Χ^2 ^= 36.9, p < 0.0001) males compared with healthy range males, and overweight (Χ^2 ^= 8.3, p = 0.0040) and obese (Χ^2 ^= 6.0, p = 0.0146) females compared with healthy range females.

### Associations between insulin and BMI, waist circumference, WHtR and FMI

Inter-quintile ranges created for BMI, waist circumference, WHtR and FMI to enable insulin concentrations to be compared between boys and girls within the same ranges of BMI, waist circumference, WHtR and FMI. Table 4 shows the mean insulin concentrations for quintiles of BMI, waist circumference and FMI for boys and girls, separately. For BMI, among boys the concentrations did not vary across quintiles 1-3 but increased progressively and among girls the concentrations did not vary across quintiles 1 and 2 but increased progressively thereafter. The mean concentrations in the first four quintiles were higher among girls compared with boys, and similar in the 5^th ^quintile, although only the difference for the 3^rd ^quintile was statistically significant.

**Table 4:** Geometric mean values of insulin concentration (pmol/L) for percentile ranges of BMI (kg/m^2^), waist circumference (cm) and fat mass index (kg/m^2^) for boys and girls

		Mean Insulin conc. (pmol/L)		
Centile ranges	BMI (kg/m^2^)	Boys	Girls	P-value of difference
0-20	≤ 18.7	49.5	58.9	0.16
21-40	18.8-20.3	50.0	55.0	0.34
41-60	20.4-21.9	49.4	70.0	< 0.01
61-80	22.0-24.3	69.9	73.8	0.53
81-100	24.4-41.4	95.6	92.0	0.74

	**Waist (cm) circumference (cm) (cm)**			

0-20	≤ 61.5	43.6	56.3	0.02
21-40	61.6-65.7	52.4	63.3	0.06
41-60	65.8-69.8	53.9	71.3	0.01
61-80	69.9-75.6	59.8	82.2	0.01
81-100	75.7-109.1	87.6	105.0	0.08

**Waist to Height Ratio (cm/cm)**				

0-20	≤ 0.37	47.6	58.0	0.06
21-40	0.38-0.39	55.2	59.9	0.37
41-60	0.40-0.41	50.2	67.3	0.01
61-80	0.42-0.44	66.9	74.9	0.41
81-100	0.45-0.64	96.0	102.1	0.49

	**FMI (kg/m^2^)**			

0-20	≤ 3.1	50.9	52.3	0.85
21-40	3.2-4.2	48.6	62.1	0.11
41-60	4.3-5.3	65.4	55.3	0.13
61-80	5.4-7.1	78.4	72.0	0.27
81-100	7.2-26.2	107.1	83.5	0.03

For waist circumference, the mean insulin concentrations were higher among girls at every quintile and became progressively higher for both boys and girls across quintiles 1-5. The differences between boys and girls were statistically significant for the 1^st^, 3^rd ^and 4^th ^quintiles. The mean insulin concentration was higher in each quintile of WHtR in girls, but was statistically significant in the 3^rd ^quintile only. For FMI, insulin concentration increased consistently across quintiles 2-5 among boys and quintiles 3-5 among girls, but in contrast with BMI and waist circumference, insulin concentration among girls was equal to or lower than the values among boys in all quintiles except the second. The differences between boys and girls were statistically significant only the 5^th ^quintile.

Pearson correlation coefficients for the associations between insulin concentration and BMI, waist circumference, WHtR and FMI among boys were 0.53, 0.49, 0.51 and 0.55, respectively, and the correlations among girls were 0.35, 0.40, 0.42 and 0.34, respectively. All were statistically significant at P < 0.001.

## Discussion

This study surveyed a representative population sample of adolescents living in a developed economy. Glucose concentration was normally distributed and was not associated with BMI category or other measures of adiposity. In contrast, insulin concentration was logarithmically distributed and was positively associated with adiposity. Insulin concentration was higher among girls than boys overall and within the same ranges of BMI, waist circumference and WHtR but tended to be higher among boys than girls within the same ranges of FMI. Using BMI or waist circumference independently to study gender differences in insulin concentration or to establish cut-points is likely to lead to erroneous conclusions. WHtR > 0.5 is a reliable option for identifying young people in need of further screening, however different cut-points should be established for boys and girls, based on BMI.

Although the definition used in this paper to identify the prevalence of elevated insulin concentration is not based on international consensus, it is one that many pediatric endocrinologists may consider reasonable. It is notable that this cut-point lies between the 85^th ^and 90^th ^percentiles for boys in the non-overweight BMI category and between the 80^th ^and 85^th ^percentiles for girls in the non-overweight BMI category. Its application showed that the prevalence of elevated insulin concentration was approximately 16-17% in this population sample. That the prevalence of elevated insulin concentrations were similar for overweight and obese girls was surprising, but may be explained by the fact that the sample only included nine obese girls, most of whose BMI values were close to the boundary between the overweight and obese categories.

Consistent with several other studies, we found that glucose concentrations were lower and insulin concentrations were higher among girls than boys [3-5,8]. Whereas the median (or mean) values of glucose concentration were similar across studies, the insulin concentration values differed markedly. Allard et al. [3] reported median glucose concentrations among 15 year olds of 5.3 mmol/L and 5.0 mmol/L and Misra et al. [8] means of 5.0 mmol/L and 4.9 mmol/L among boys and girls, respectively. The median values for the SPANS data reported here were 4.7 mmol/L and 4.5 mmol/L among boys and girls, respectively. With regard to insulin concentrations, Allard et al. [3] reported median insulin concentrations of 38.7 pmol/L and 46.4 pmol/L, Misra et al. [8] reported means of 96.0 pmol/L and 118.8 pmol/L, Wennlöf et al. [4] reported medians of 55.9 pmol/L and 48.0 pmol/L among boys and girls, respectively, and the SPANS study reported 54 pmol/L among both boys and girls. That is, the SPANS values were similar to those of Allard et al. and Wennlöf et al., but approximately half those of Misra et al.

Unfortunately, Allard et al. did not provide summary statistics for BMI for the Canadian sample and the mean BMI values of Misra et al.'s and Wennlöf et al.'s, samples were only slightly lower than those of the SPANS sample (data not shown). The small differences in adiposity between the samples would not appear to account for the very large differences between Misra et al.'s findings and the other studies, but it is plausible that ethnic differences between the samples may account for them [16]. Whereas Allard et al., Wennlöf et al. and SPANS sampled from primarily Europid populations, Misra et al. sampled from an Asian Indian population. Despite the differences between the SPANS and Misra et al.'s findings, the correlations between insulin concentration and BMI reported by Misra et al. (r = 0.62 for boys and r = 0.39 for girls) were similar to those reported here.

The SPANS study had several limitations. First, participation rates were lower than would have been preferred, but we reported evidence that the sample was representative of the study population [17]. Second, the obese BMI category only included nine girls with a small range of BMI values, although the prevalence of obesity among girls in the biomarker sub-study was not statistically different from that in the study as a whole. The low number of girls may have influenced the finding that insulin concentrations at the 85^th ^and 95^th ^centiles among all boys and obese boys, were much higher than among girls. Similarly, the small number of obese girls may explain why the prevalence of elevated insulin concentration was relatively low among obese girls, compared with obese boys. Data from a greater number of obese girls with a greater range of BMI values would enhance the quality of the present findings, as would the collection of family history of diabetes.

## Conclusion

This study provides a useful source of insulin reference values for clinicians collected on free-living adolescents and has characterized the nature of the relationship between insulin concentration and adiposity among adolescents. Although the definition of elevated insulin used in this study is somewhat arbitrary, the data suggest that elevated insulin concentrations are associated with excess adiposity in adolescents. Longitudinal studies are required to determine the long term sequela of elevated insulin from a relatively young age

## Competing interests

The authors declare that they have no competing interests.

## Authors' contributions

EDW was responsible for study design, data collection and interpretation and preparation of manuscript. CC provided advice to study coordinators, data interpretation and review of the manuscript. ADO was responsible for study management, study design, interpretation of results and review of the manuscript. LH oversaw data collection and provided review of the manuscript. RA and TD provided statistical analysis and reviewed the manuscript. All have read and agree with the final manuscript.

## Pre-publication history

The pre-publication history for this paper can be accessed here:

http://www.biomedcentral.com/1471-2431/10/58/prepub
